# *Pseudomonas aeruginosa* Pyocyanin Activates NRF2-ARE-Mediated Transcriptional Response via the ROS-EGFR-PI3K-AKT/MEK-ERK MAP Kinase Signaling in Pulmonary Epithelial Cells

**DOI:** 10.1371/journal.pone.0072528

**Published:** 2013-08-27

**Authors:** Ying Xu, Chaohui Duan, Zhizhou Kuang, Yonghua Hao, Jayme L. Jeffries, Gee W. Lau

**Affiliations:** 1 Department of Pathobiology, University of Illinois at Urbana-Champaign, Urbana, Illinois, United States of America; 2 Laboratory of Clinical Immunology, Sun Yat-Sen Memorial Hospital, Sun Yat-Sen University, Guangzhou, Guangdong, People’s Republic of China; University of Sherbrooke, Canada

## Abstract

The redox-active pyocyanin (PCN) secreted by the respiratory pathogen *Pseudomonas aeruginosa* generates reactive oxygen species (ROS) and causes oxidative stress to pulmonary epithelial cells. Nuclear factor (erythroid-derived 2)-like 2 (NRF2) confers protection against ROS-mediated cell death by inducing the expression of detoxifying enzymes and proteins via its binding to the cis-acting antioxidant response element (ARE). However, a clear relationship between NRF2 and PCN-mediated oxidative stress has not been established experimentally. In this study, we investigated the induction of NRF2-ARE response by PCN in the pulmonary epithelial cells. We analyzed the effect of PCN on NRF2 expression and nuclear translocation in cultured human airway epithelial cells, and in a mouse model of chronic PCN exposure. NRF2-dependent transcription of antioxidative enzymes was also assessed. Furthermore, we used inhibitors to examine the involvement of EGFR and its downstream signaling components that mediate NRF2-ARE-activation in response to PCN. PCN enhances the nuclear NRF2 accumulation and activates the transcription of ARE-mediated antioxidant genes. Furthermore, PCN activates NRF2 by inducing the EGFR-phosphoinositide-3-kinase (PI3K) signaling pathway and its main downstream effectors, AKT and MEK1/2-ERK1/2 MAP kinases. Inhibition of the EGFR-PI3K signaling markedly attenuates PCN-stimulated NRF2 accumulation in the nucleus. We demonstrate for the first time that PCN-mediated oxidative stress activates the EGFR-PI3K-AKT/MEK1/2-ERK1/2 MAP kinase signaling pathway, leading to nuclear NRF2 translocation and ARE responsiveness in pulmonary epithelial cells.

## Introduction

*Pseudomonas aeruginosa (PA)* is an important bacterial pathogen causing acute nosocomial infections in immunocompromised patients, and chronic recurring lung infections in patients with cystic fibrosis (CF) or non-CF bronchiectasis [1], [2]. The ability of *PA* to cause diseases is due, in part, to its ability to form biofilms and to release a large cache of virulence factors, including exoproteases, phospholipases, hemolysin, rhamnolipids and phenazines [3]–[6]. Among the phenazines, pyocyanin (PCN), a blue redox-active secondary metabolite, is an important virulence factor in the pathogenesis of pseudomonal lung diseases. PCN has been recovered in varying concentrations from trace quantities to concentrations up to 100 µM (27 µg/ml sputum) in pulmonary secretions of bronchiectatic patients infected by *PA* [7], [8], as well as in ear secretions of *PA*-mediated chronic suppurative otitis media [9]. Additionally, chronic instillation of PCN causes goblet cell hyperplasia and metaplasia (GCHM), fibrosis, destruction of alveolar airspace and influx of neutrophils in mouse lungs, pathophysiological features that resemble those observed in the CF and non-CF bronchiectatic lungs [10], [11].

The virulence effects of PCN are mediated by its ability to induce the formation of ROS, which oxidatively damage pulmonary epithelial cells [12]–[15]. Upon entry into host cells, PCN undergoes a non-enzymatic reduction by NADPH or other reducing agents and rapidly reacts with molecular oxygen to produce superoxide [16] and, by dismutation, hydrogen peroxide [17]. Superoxide could react with nitric oxide to form highly toxic reactive nitrogen species that damage DNA, proteins and phospholipids and modulate host immune response [18].

The endogenous cellular antioxidant system consists of antioxidant enzymes such as superoxide dismutase, glutathione peroxidase, glutathione reductase, catalase, heme oxygenase-1 [19], and the phase 2 detoxification enzymes represented by glutathione-S-transferase (GST) isozymes and NADP(H):quinone oxidoreductase (NQO1) [20]. These proteins cope with the oxidative burden and limit potential toxicity by ROS. However, excessive ROS may perturb the balance of the oxidation-reduction equilibrium, leading to oxidative stress. NRF2, a member of the Cap’n’Collar family of bZIP transcription factors, is critical in cytoprotection of airway epithelia by transcriptional induction of genes encoding ROS detoxifying enzymes and proteins [21], [22]. In normal cells, NRF2 is predominantly sequestered in the cytoplasm in association with KEAP1, an actin-binding cytoskeletal protein [23]. Oxidative insults cause dissociation of the NRF2-KEAP1 complex, triggering the translocation of NRF2 from the cytoplasm to the nucleus [23]. Nuclear NRF2 binds to the cis-acting antioxidant response element (ARE) and upregulates the transcription of genes critical for cellular antioxidant processes [21], [24]–[26].

Despite many years of studies of PCN, a clear relationship between NRF2 and PCN-mediated oxidative stress has not been established experimentally. Because the lung is the major target of oxidative injuries [27], we examined the induction of NRF2 by PCN using both the human lung epithelial cell lines and a mouse model of chronic PCN exposure. We demonstrate that PCN activates NRF2 through the ROS-inducible EGFR-PI3K cellular signal transduction pathway and its downstream effectors.

## Materials and Methods

### PCN

PCN was purchased from Sigma Chemical (#R9532). Chemically-synthesized PCN is preferred over PCN purified from *PA* cultures to eliminate any contaminants (e.g. LPS, CpG DNA, etc), which may cause lung injuries. PCN was resuspended to 1 µg/ml in sterile H_2_O.

### Antibodies

Primary monoclonal and polyclonal antibodies were purchased from commercial suppliers. The following antibodies and dilutions were used for western blotting analyses. Santa Cruz Biotechnology: NRF2 (sc-722, dilution 1∶2000), EGFR (sc-03, dilution 1∶1000), phosphorylated p-EGFR (sc-101668, dilution 1∶500), AKT1 (sc-5298, dilution 1∶1000) and Histone H3 (sc-10809, dilution 1∶1000); Cell Signaling Technology: p-AKT (4060, dilution 1∶1000), MEK1/2(2338, dilution 1∶2000), pMEK1/2 (9154, dilution 1∶2000), ERK1/2(4348, dilution 1∶1000), p-ERK1/2 (9101, dilution 1∶1000), and GAPDH (2118, dilution 1∶4000). Finally, goat-anti rabbit IgG HRP-conjugated secondary antibody (Santa Cruz Biotechnology sc-2030) was used at a dilution of 1∶4000.

### Cell Cultures

**Figure 1:**
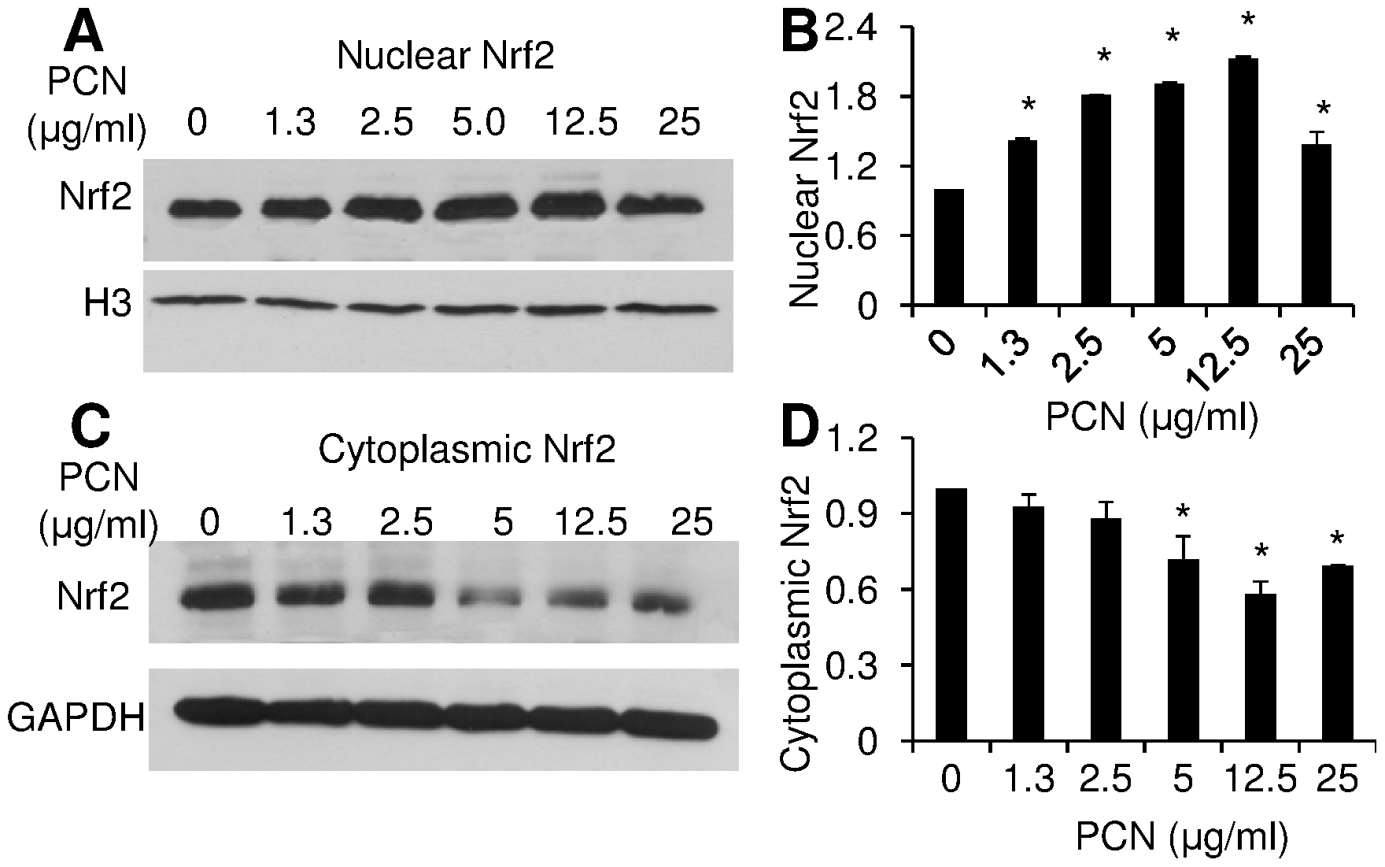
PCN induces nuclear accumulation of NRF2 in a concentration-dependent manner. A549 cells were exposed to various concentrations of PCN for 12 hr. Total nuclear and cytoplasmic proteins were harvested for Western blot using anti-NRF2 monoclonal antibody. The same membrane was stripped and probed with antibody against nuclear protein Histone H3 or cytoplasmic protein GAPDH for loading controls. The experiments were repeated three times with similar results. The western blots from one typical experiment are shown. Densitometry data represent the mean ± SD from all three experiments. *p<0.05 when PCN-exposed cells were compared with the control cells exposed to same volume of sterile water. (**A**) Nuclear NRF2 after 12 hr of exposure to indicated concentrations of PCN. (**B**) Densitometry analysis of the nuclear NRF2. (**C**) Cytoplasmic NRF2 after 12 hr of exposure to PCN. (**D**) Densitometry analysis of the cytoplasmic NRF2.

The human alveolar type-II epithelial cell line A549 was purchased from the American Type Culture Collection (ATCC) (Manassas). A549 cells are routinely used in the study of oxidative stress, including deciphering the protective roles of NRF2 [28]–[31]. Cells were cultured in RPMI-1640 supplemented with 10% fetal bovine serum in 5% CO_2_. Epithelial cells that reached 70% conﬂuency were serum-starved for 24 hr before exposure to PCN. As controls, cells were exposed to the same volume of sterile H_2_O. For example, in Figure 1, control cells were exposed to 25 µl H_2_O per ml of RPMI-1640 culture medium.

### Measurement of Intracellular ROS

Total ROS levels in A549 cells treated with PCN or PBS were measured with the Oxiselect™ in vitro ROS/RNS assay kit (Cell Biolabs, Inc.), following the manufacturer’s instruction. The assay uses the specific ROS/RNS probe dichlorodihydrofluorescin DiOxyQ (DCFH-DiOxyQ). The DCFH-DiOxyQ probe is first primed with a quench removal reagent, and subsequently stabilized in the highly reactive DCFH form. ROS and RNS species react with DCFH, which then rapidly oxidizes to the highly fluorescent 2′, 7′-dichlorodihydrofluorescein (DCF). Fluorescence intensity is proportional to the total ROS/RNS levels within the sample. The DCFH-DiOxyQ probe can react with hydrogen peroxide (H_2_O_2_), peroxyl radical (ROO⋅), nitric oxide (NO), and peroxynitrite anion (ONOO^−^), allowing for measurement of the total free radical population within a sample. After the assay, the relative fluorescence of the samples and the standards were read at 480 nm excitation/530 nm emissions using SpectraMaxEM microplate reader (Molecular Devices). The concentration of the total ROS was calculated using the 2′, 7′-dichlorodihydrofluorescein standard curve.

### Immunofluorescence of A549 Cells

A549 cells were exposed to PCN (12.5 µg/ml) for the indicated time intervals. Immunofluorescence analysis was performed using primary anti-NRF2 antibody, followed by goat anti-Rabbit DyLight® 488-conjugated secondary antibody (Abcam ab96895, dilution 1∶100). Slides were mounted using DAPI, and the subcellular localization of NRF2 was observed using a confocal fluorescence microscope (Zeiss Axiovert 200 M). Quantitative analyses of mean density of fluorescence in nuclei were performed with AxioVision Rel. 4.8 software (Carl Zeiss MicroImaging, LLC). The mean fluorescence density in nuclei was quantified from 20 microscopic fields (∼5–10 cells/field) of each time point, and then compared to the control cells exposure to same volume of sterile water (12.5 µl H_2_O/ml RPMI-1640).

### Ethics Statement

The animal study was carried out in strict accordance with the recommendations in the Guide for the Care and Use of Laboratory Animals of the National Institutes of Health. The protocol was approved by the Institutional Animal Care and Use Committee (IACUC) at the University of Illinois at Urbana-Champaign (Protocol Number: 10144).

### Mouse Model of Chronic PCN Exposure

C57BL6 mice (6 week old, groups of 8) were housed in positively ventilated micro-isolator cages with automatic recirculating water, located in a room with laminar, high efficiency particle accumulation-filtered air. The animals received autoclaved food, water and bedding. PCN (25 µg in 50 µl of sterile H_2_O) was intranasally inoculated into the lungs of C57BL6 mice anaesthetized with isoﬂurane once a day for 3 weeks. Control mice were exposed to 50 µl of sterile water. These time points were chosen based on our previous studies demonstrating PCN-induced GCHM in C57BL6 mice, where clear differences in the lung histopathology, cytokine and profile of immune cells can be detected between the PCN-treated and control mice [10], [11].

### Immunofluorescence Evaluation of Mouse Lung Tissues

After PCN exposure, mouse lungs were collected for immunofluorescence assays. Brieﬂy, a cannula was inserted in the trachea, and the lung was instilled with 10% neutral-buffered formalin at a constant pressure (25 cm H_2_O). The trachea was ligated, and the inﬂated lung was immersed in 10% neutral buffered formalin for 24 hr before embedded in paraffin wax. Paraffin-embedded sections (4–8 µm thickness) were stained with Periodic Acid-Schiff’s reagent (PAS) for mucins. For immunofluorescence assay, lung sections were stained with primary anti-NRF2 antibody listed above (1∶100 dilution) and visualized by the goat anti-Rabbit DyLight® 488-conjugated secondary antibody (Abcam ab96895, dilution 1∶100). Subcellular localization of NRF2 was observed using a confocal fluorescent microscope (Zeiss Axiovert 200 M). Quantitative analyses of the NRF2-positive nuclei in bronchial epithelial cells were performed with the AxioVision Rel. 4.8 software from 5 lung sections per each mouse (n = 8 mice) exposed to PCN versus sterile water control.

### Quantitative Real-time Polymerase Chain Reaction (qRT-PCR) Analysis

A549 cells were cultured in 6-well plates and stimulated with 0, 5.0, or 12.5 µg/ml of PCN for 12 hr in the presence or absence of various inhibitors. Total RNA was extracted using the RNeasy Mini Kit (Qiagen # 74104) according to the manufacturer’s instructions. Equal amount of total RNA (2 µg) was reverse transcribed into cDNA using oligo(dT) primers and SuperScript III reverse transcriptase (Invitrogen). After the reverse transcription reaction, the first-stranded cDNA was then diluted and used in each subsequent PCR reaction. qRT-PCR was performed on a 7900 HT real-time PCR system by using 1 µl of cDNA in the presence of Taqman primers synthesized by the Applied Biosystems (Grand Island, New York). The relative expression of each gene was normalized to *GAPDH* to give a relative expression level. The primer information is provided below: *NQO1*: forward primer: 5′-AGTCCCTGCCATTCTGAAAGG-3′, reverse primer: 5′-GCGTAAGTGTAAGCAAACTCTCCTA- 3′, γ-*GCSh*: forward primer: 5′-GGGAGTGATTTCTGCATCTGTAGAT-3′; reverse primer: 5′-TGAGTCATATCGGGATTTACTGATCCT-3′, The primers for the *GAPDH* gene are propriety information belonging to the Applied Biosystems. The Assay ID for the primers is Hs99999905_m1.

### Crude Plasma Membrane Purification for Enrichment of EGFR

Western blot analysis of EGFR was performed using crude plasma membrane proteins of A549 cells. Crude plasma membranes were prepared as follows [32]. Briefly, A549 cells previously treated with PCN were washed with PBS and detached with a scraper. Detached cells were resuspended in lysis buffer (20 mM Tris-HCl, 200 mM sucrose, 5 mM EDTA and 1∶100 protease inhibitor cocktail) and homogenized with 25 strokes of a dounce homogenizer. The homogenates were centrifuged at 6,000 g for 10 min to remove nuclei and unbroken cells. The supernatants were centrifuged at 34,000 g for 60 min at 4°C to pellet the membranes. The remaining supernatants were kept as the cytoplasmic fraction.

### Western Blotting Analyses

For Western blotting analyses, proteins were separated by 8% or 10% SDS-PAGE and transferred to PVDF membranes (Bio-Rad). Membranes were probed with individual antibodies against NRF2, EGFR, pEGFR, AKT, pAKT, MEK1/2, pMEK1/2, ERK1/2 or pERK1/2 as described above. The immune blots were visualized using the ECL Western Blotting Detection System (Amersham Biosciences) and Kodak BIOMAX (Kodak) X-ray films. Western blots were quantified semi-quantitatively by densitometry analyses and normalized based on the loading controls.

### Inhibitor Studies

After reaching 70% conﬂuency, A549 cells were serum-starved for 24 hr, and exposed to the NRF2 inhibitor trigonelline hydrochloride (Sigma), EGFR inhibitor AG1478 (Calbiochem), PI3K inhibitor LY294002 (Calbiochem), ERK1/2 inhibitor 3-(2-Aminoethyl)-5-((4-ethoxyphenyl)methylene)-2,4-thiazolidinedione (Sigma), AKT Inhibitor VIII trifluoroacetate salt hydrate (Sigma), and/or the antioxidant glutathione (GSH) (Sigma) at indicated concentrations. After 90 min of exposure, PCN (12.5 µg/ml) was added for 12 hr. Control cells were incubated with 12.5 µl of sterile H_2_O. The amounts of NRF2 in the nuclei of A549 cells were determined using the specific anti-NRF2 antibody.

### Statistical Analysis

Quantitative data were expressed as the mean ± standard error. Statistical significance comparisons for samples with equal variances were determined using the parametric Student’s *t* test for two unpaired samples and the parametric one-way ANOVA for 4 independent grouped samples. A significant difference was considered to be *p*<0.05.

## Results

### PCN Increased ROS Production in Pulmonary Epithelial Cells

To determine whether PCN significantly alters ROS and causes oxidative stress to pulmonary epithelial cells, we evaluated total ROS levels in the A549 cells. Cells were treated with PCN in four different clinically-relevant concentrations [7], [8] between 2.5 and 25 µg/ml for 12 hr. ROS levels were elevated in a concentration-dependent manner by up to 3.02-fold in A549 cells (Figure S1A). Trypan blue exclusion assays indicate that >97% of cells remained viable (Figure S1B). These results indicate that PCN rapidly generates cytotoxic ROS in pulmonary epithelial cells.

### PCN Induces Nuclear NRF2 Accumulation in Concentration and Time-dependent Manners

Previously, it has been reported that, hyperoxia-induced NADPH oxidase and ROS-mediated signaling controls NRF2 translocation from the cytoplasm to the nucleus [26]. Because PCN induces robust levels of ROS in A549 cells, we examined whether PCN-generated ROS trigger nuclear translocation of NRF2 by Western blotting analyses. A549 cells were exposed to various concentrations of PCN for 12 hr, and nuclear and cytoplasmic NRF2 were monitored using specific antibodies. At five concentrations between 1.3 and 25 µg/ml, PCN significantly increased the nuclear NRF2 accumulation, with maximal increase of 113% in cells exposed to 12.5 µg/ml of PCN (Figure 1A, 1B). Importantly, increasing levels of nuclear NRF2 was associated with decreasing levels of NRF2 expression in cytoplasm (Figure 1C, 1D). For example, the expression of NRF2 in cytoplasm decreased by 42% in cells exposed to 12.5 µg/ml PCN. These results suggest that PCN stimulates the translocation of NRF2 from the cytoplasm to the nuclei of airway epithelial cells.

**Figure 2:**
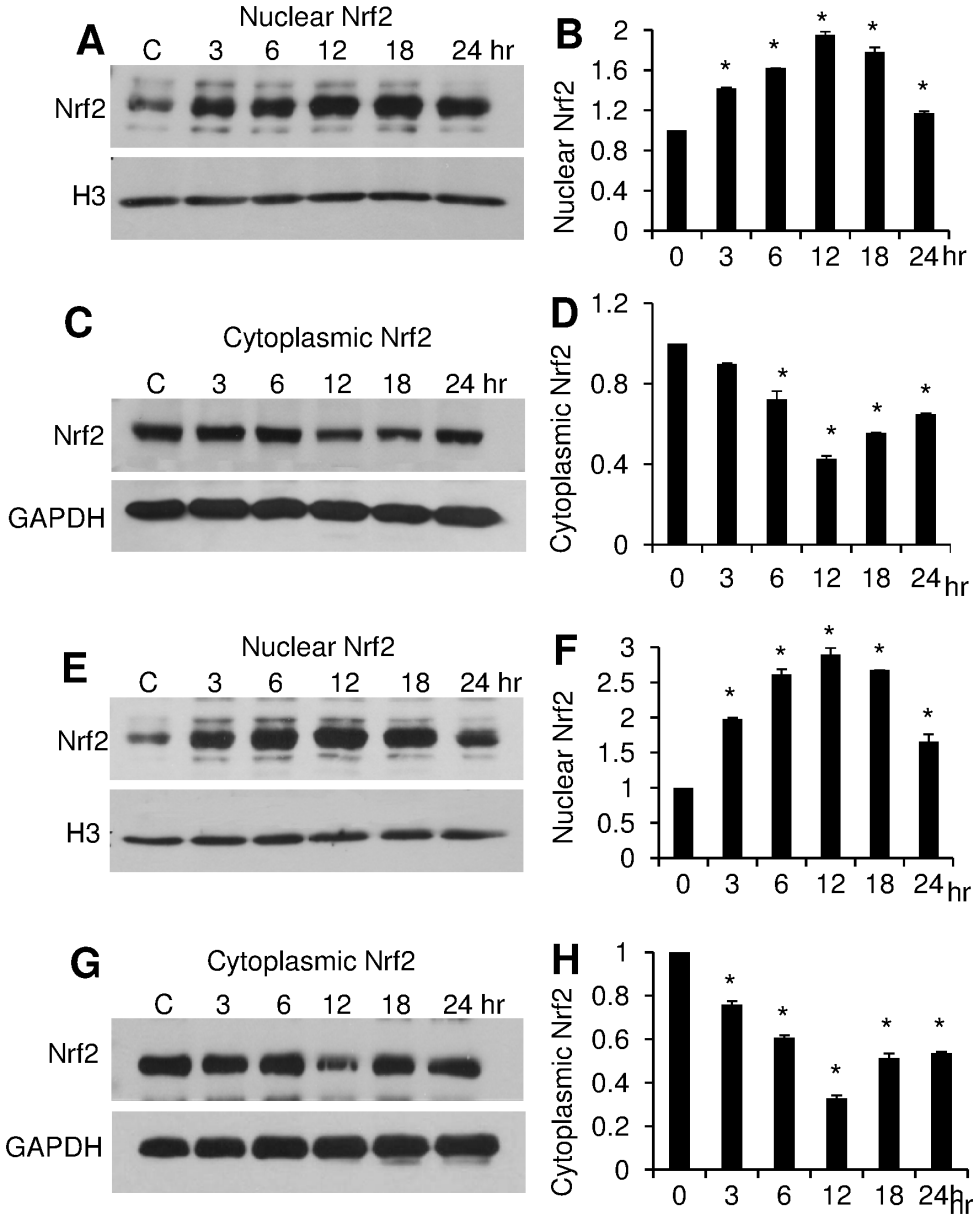
PCN induces nuclear NRF2 accumulation in a time-dependent manner. A549 cells were exposed to 5.0 µg/ml (**A–D**) and 12.5 µg/ml (**E–H**) of PCN, respectively, for indicated time intervals. Total nuclear and cytoplasmic proteins were harvested for Western blot using an anti-NRF2 monoclonal antibody. Histone H3 and GAPDH were used as loading controls. The experiments were repeated three times with similar results. The western blots from one typical experiment are shown. Densitometry data represent the mean ± SD from all three experiments. *p<0.05 when PCN-exposed cells were compared against the control cells exposed to same volume of sterile water. (**A–B**) Western blots and densitometry analyses of nuclear NRF2 after 24 hr of exposure to 5.0 µg/ml PCN. (**C–D**) Western blots and densitometry analyses of cytoplasmic NRF2 after 24 hr of exposure to 5.0 µg/ml PCN. (**E–F**) Western blots and densitometry analyses of nuclear NRF2 after 24 hr of exposure to 12.5 µg/ml PCN. (**G–H**) Western blots and densitometry analyses of cytoplasmic NRF2 after 24 hr of exposure to 12.5 µg/ml PCN.

To further verify this result, we examined the dynamics of NRF2 in a time-dependent manner following exposure to PCN. Cells were exposed to PCN in the concentrations of 5.0 and 12.5 µg/ml, respectively, which have been shown to induce higher nuclear accumulation of NRF2 (Figure 1). At 5 µg/ml concentration, PCN markedly stimulated NRF2 translocation from the cytoplasm to the nucleus as early as 3 hr after exposure (1.5 to 2-fold), reached its peak at 12 hours (2 to 3-fold) (Figure 2A–D). Again, the accumulation of NRF2 in nuclei was correlated with a decrease (1 to 1.5-fold) in the cytoplasm. At 12.5 µg/ml concentration, PCN induced a more robust nuclear localization and corresponding cytoplasmic depletion of NRF2 (Figure 2E–H). Collectively, these results suggest that PCN-mediated ROS trigger the translocation of NRF2 from the cytoplasm to the nucleus in concentration and time-dependent manners.

### PCN Stimulates NRF2 Translocation from the Cytoplasm to the Nuclei of A549 Cells

**Figure 3:**
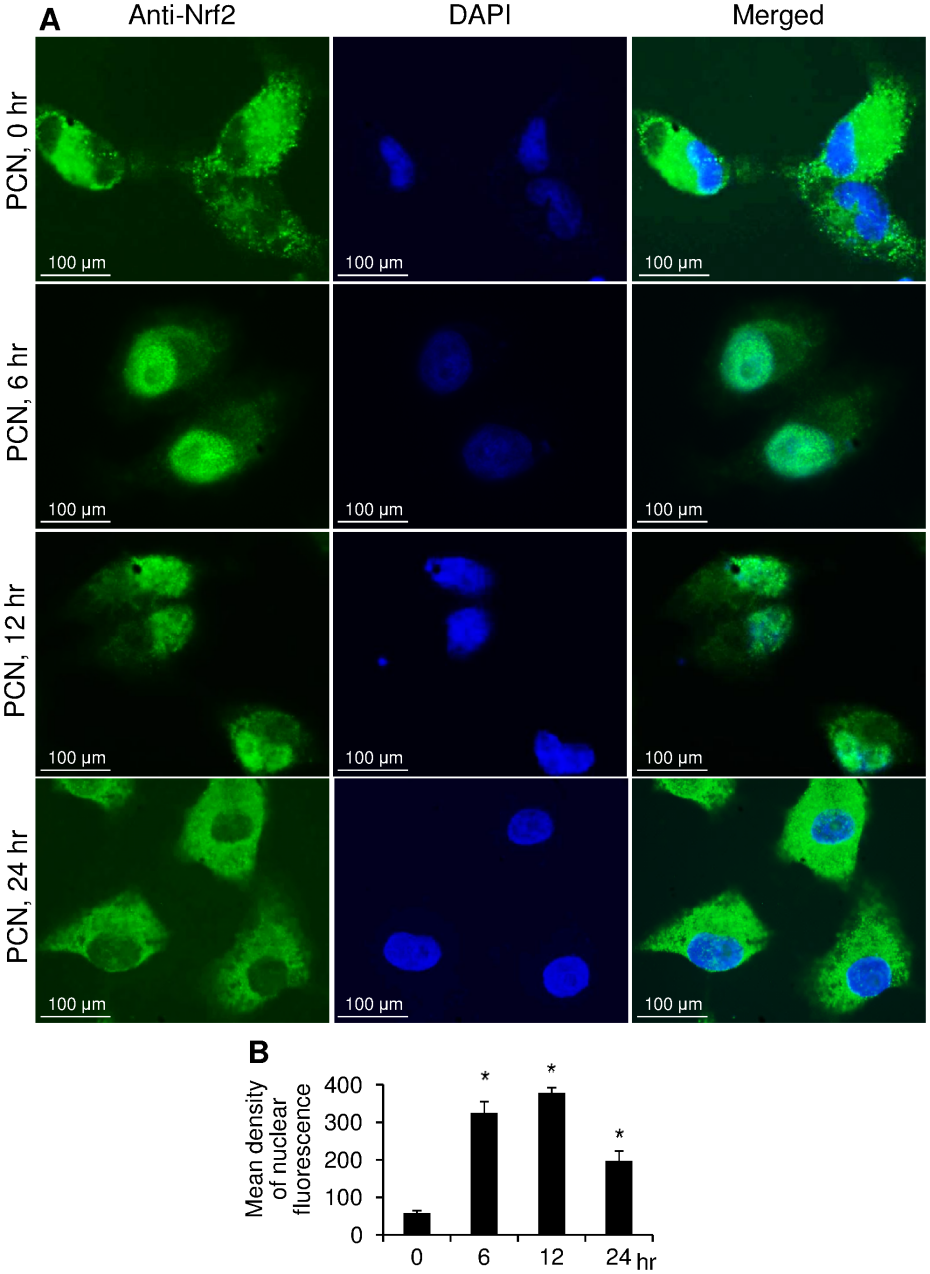
Immunofluorescence analysis of PCN-mediated NRF2 nuclear translocation. (**A**) A549 cells were exposed to 12.5 µg/ml PCN for the indicated time intervals. NRF2 was probed with a primary anti-NRF2 antibody and visualized with a goat anti-Rabbit DyLight® 488-conjugated secondary antibody. Nuclei were stained with DAPI. Images were photographed using confocal fluorescence microscopy. (**B**) Quantification of nuclear NRF2 in control versus PCN-exposed cells.

Next, we utilized fluorescence microscopy to further confirm the PCN-induced nuclear translocation of NRF2 from the cytoplasm. A549 cells were exposed to PCN (12.5 µg/ml) for 0, 6, 12, 24 hr, and visualized with specific anti-Nrf2 antibody and DAPI (Fig. 3A). Immediately after exposure to PCN (12.5 µg/ml) (0 hr), NRF2 was predominantly localized in the cytoplasm of A549 cell. By 6 hr and 12 hr after exposure to PCN, NRF2 was predominantly localized to the nuclei. Semi-quantitative analysis using the AxioVision Rel. 4.8 software indicated that PCN significantly increased the accumulation of NRF2 in the nuclei (mean fluorescence density in nucleus), with maximal increase by 6.5-fold (Figure 3B) after 12 hr exposure. However, this translocation process was transient, and by 24 hr, the immunofluorescence signals in nuclei were weakened. At this stage, the expression of the cytoplasmic NRF2 regained its dominance over its expression in nuclei, suggesting that NRF2 had relocalized to the cytoplasm, which correlates with the Western blotting results shown in Figures 1 and 2. Collectively, these results suggest that PCN-mediated ROS triggers the translocation of NRF2 from the cytoplasm to the nucleus.

### PCN Induces Nuclear Translocation of NRF2 during Goblet Cell Hyperplasia and Metaplasia in Mouse Airways

**Figure 4:**
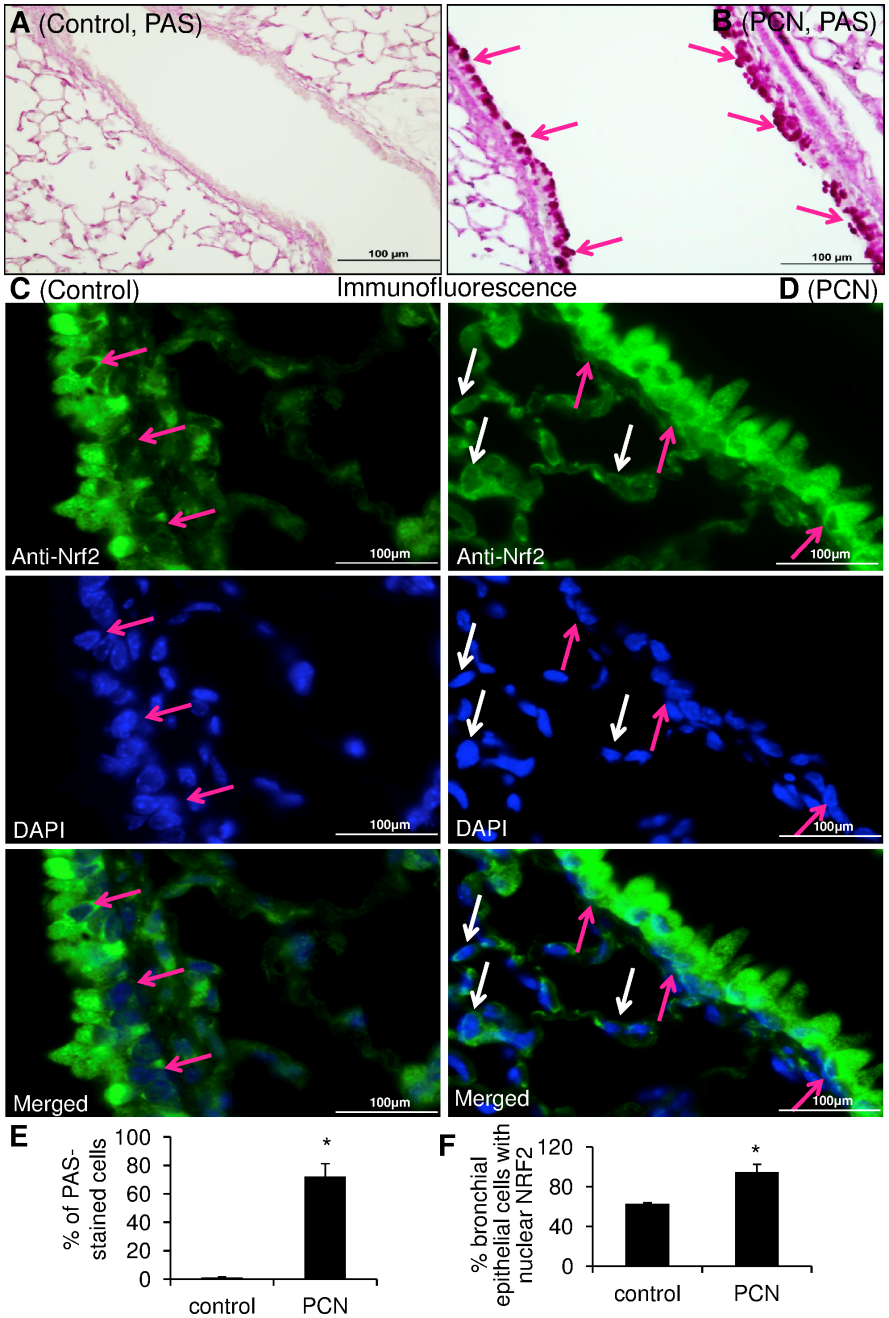
Nuclear translocation of NRF2 is increased in PCN-mediated goblet cell hyperplasia in mouse airways. Mouse lungs (groups of 12) were exposed to sterile water control (**A** and **C**) or PCN (**B** and **D**). (**A–B**) Lung sections were PAS stained for mucins (red color) and photographed with a light microscope. (**C–D**) Lung sections were stained with an anti-NRF2 primary antibody and visualized with a goat anti-Rabbit DyLight® 488-conjugated secondary antibody. Nuclei were stained with DAPI. Images were photographed using confocal fluorescence microscope. (**E**) Quantification of PAS-positive cells in control versus PCN-exposed airways. (**F**) Quantification of goblet cells with nuclear NRF2 in control versus PCN-exposed airways.

Most recently, we and others have shown that PCN induces the goblet cell hyperplasia and metaplasia and mucus hypersecretion in mouse airways partly by activating the EGFR signaling pathway [11], [15]. MAP kinases acting downstream of the EGFR have been shown to regulate NRF2 function [33], [34]. The abovementioned observations suggest that the expression and nuclear localization of NRF2 may be altered during PCN-mediated GCHM. As shown in Figure 4, control lungs exposed to sterile H_2_O remained normal, with only basal levels of mucus (Figure 4A, 4E) and nuclear NRF2 (Figure 4C; Figure S2A). In contrast, after 3 weeks of exposure to PCN, large airways of C57BL6 mice developed GCHM and mucus hypersecretion as evident by PAS staining (Figure 4B, 4E). Immunofluorescence microscopy of PCN-exposed airways reveals enhanced NRF2 fluorescence signal in the nuclei of goblet cells that obscures the DAPI staining (Figure 4D, 4F; Figure S2B). Importantly, the nuclei of alveolar epithelial cells remained largely clear of NRF2 (white arrows). These results suggest that PCN induces nuclear translocation of NRF2 in the epithelial cells that are undergoing trans-differentiation to goblet cells in airways.

### Increased Nuclear Localization of NRF2 Enhances the Transcription of Antioxidant Genes

**Figure 5:**
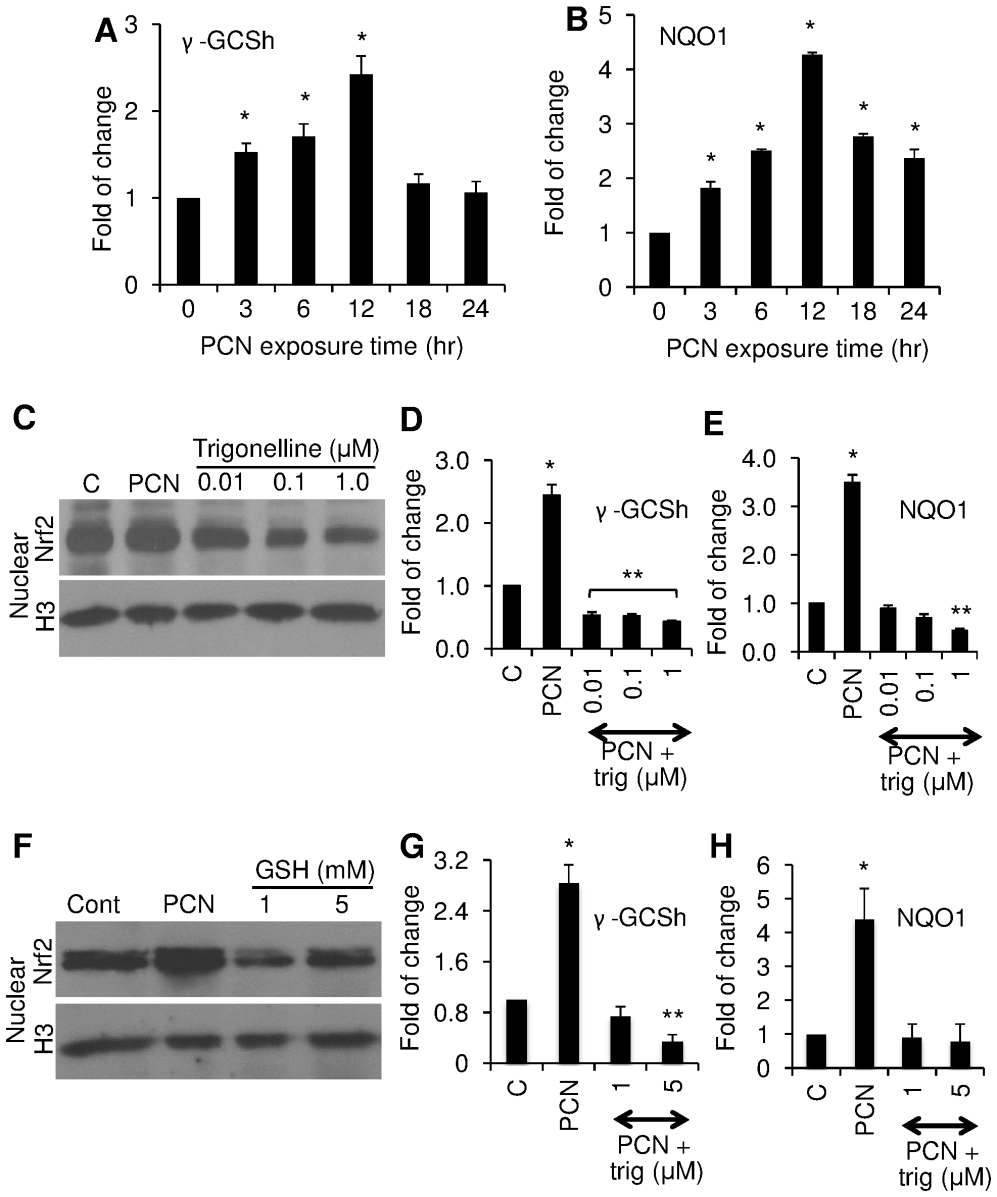
Translocation of NRF2 into nuclei correlates with the induction of *γ-GCSh* and *NQO1* gene expression. The expression of *γ-GCSh* and *NQO1* genes were quantified by qRT-PCR using total RNA extracted from A549 cells after exposure to 12.5 µg/ml PCN in the presence or absence of trigonelline or GSH for the indicated time intervals and concentrations. (**A**) qRT-PCR analyses of *γ*-*GCSh* gene expression. (**B**) qRT-PCR analyses of *NQO1* gene expression. (**C**) Trigonelline blocked the PCN-induced nuclear translocation of NRF2. (**D–E**) qRT-PCR of *γ*-*GCSh* and *NQO1* expression in the presence of trigonelline. (**F**) GSH blocked PCN-induced nuclear translocation of NRF2. (**G–H**) qRT-PCR of *γ*-*GCSh* and *NQO1* expression in the presence of 1 or 5 mM GSH. qRT-PCR was performed in triplicates in three independent experiments. Results from one typical experiment are shown. *p<0.05 when cells that were exposed to PCN were compared to control cells exposed to the same volume of sterile water. **p<0.05 when cells that were exposed to AG1478 or LY294002 or AG1478 plus LY294002, or AKT and ERK inhibitors were compared to control cells exposed to PCN alone. Western blots were performed in three independent experiments. Results from one typical experiment are shown.

It is well established that activated NRF2 in the nucleus can bind to ARE by forming a heterodimer with other transcription factors, such as MAF, and regulate the transcription of antioxidant genes harboring the ARE cis-acting element [22]. To evaluate the effects of the nuclear accumulation of NRF2 induced by PCN, we monitored the expression of the ARE-containing antioxidant genes γ-GCSh and NQO1. The γ-GCS catalyzes the rate limiting de novo biosynthesis of glutathione (GSH) whereas NQO1 is a cytosolic protein that reduces and detoxifies quinones and their derivatives, thus protecting cells against redox cycling and oxidative stress [20]. The transcription level of both *γ-GCSh* (Figure 5A) and *NQO1* (Figure 5B) genes increased 2.4 and 4.3-fold, respectively, at 12 hr post-PCN exposure. The peak expression of both *γ-GCSh* and *NQO1* coincides with peak nuclear localization of NRF2 induced by PCN (Figure 2). In contrast, the expression of *γ-GCSh* and *NQO1* decreased at 18 and 24 hr, corresponding to decreasing levels of nuclear NRF2 (Figure 2).

To confirm that PCN indeed activates the transcription of both *γ-GCSh* and *NQO1* genes through ROS-induced NRF2, we used the NRF2 inhibitor trigonelline and the antioxidant GSH to determine whether these two compounds would inhibit the transcription of *γ-GCSh* and *NQO1* genes by PCN-induced NRF2. As shown in Figure 5C, trigonelline at concentrations of 0.01 µM and higher significantly inhibited the nuclear localization of NRF2, with corresponding decrease in the transcription of both *γ-GCSh* and *NQO1* genes (Figure 5D, 5E). Similarly, GSH at 1 and 5 mM significantly reduced the nuclear localization of NRF2 (Figure 5F), with corresponding decrease in the transcription of both *γ-GCSh* and *NQO1* genes (Figure 5G, 5H). Collectively, these data suggests that PCN induces nuclear NRF2 translocation through generation of ROS, which subsequently upregulates the transcription of antioxidant genes to detoxify oxidative stresses generated by this redox-active toxin.

### PCN Activates the EGFR-PI3K-AKT/MEK1/2-ERK1/2 MAP Kinase Signaling Pathway to Induce Nuclear NRF2 Translocation

**Figure 6:**
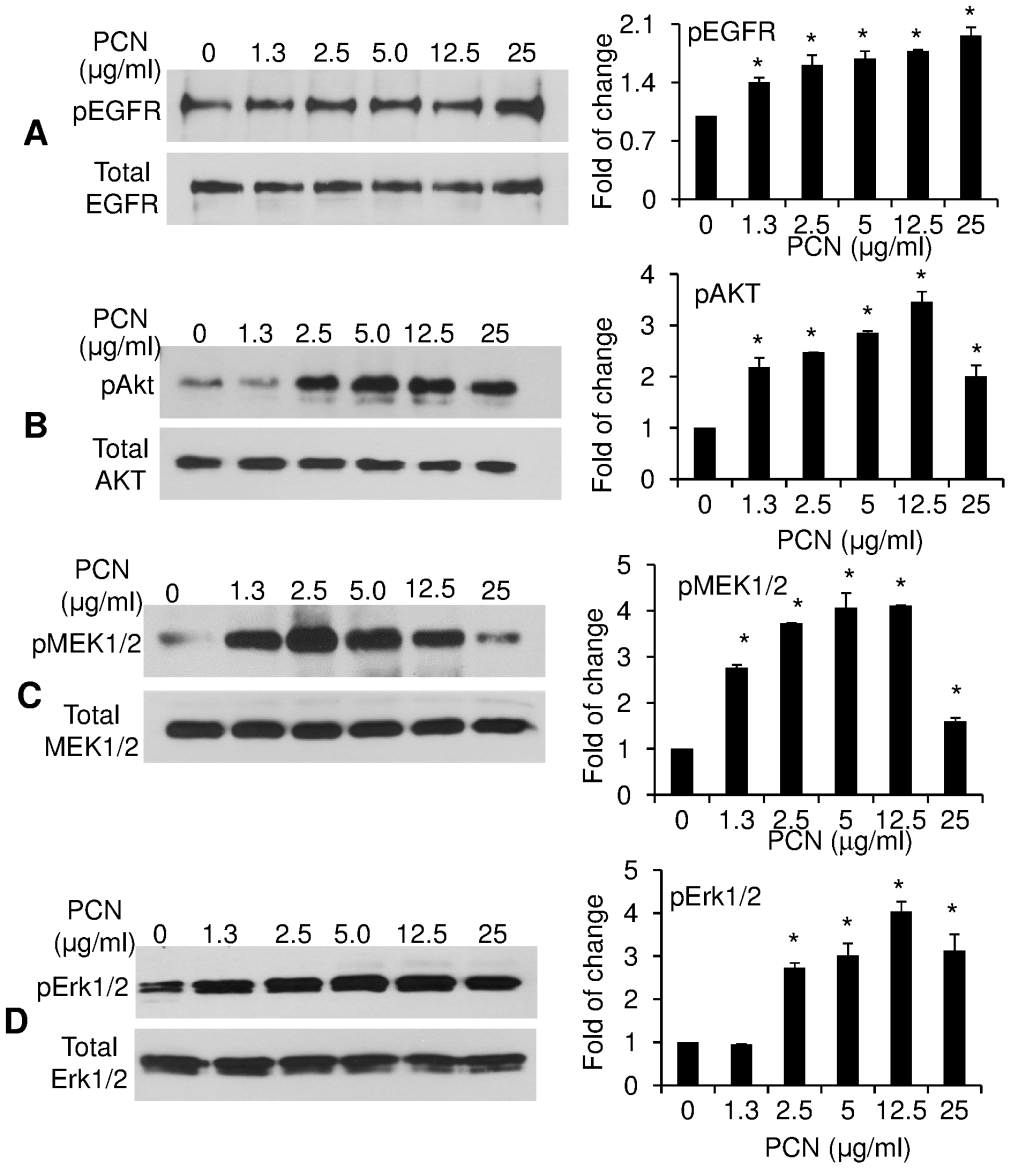
PCN activates the EGFR-PI3K-AKT/MEK1/2-ERK1/2 MAP kinase signaling pathway that positively regulates NRF2 during oxidative stress. Cytoplasmic or crude plasma membrane proteins of A549 cells previously exposed to PCN or sterile H_2_O were probed with anti-phospho–specific antibodies. (**A–D**) PCN induces the phosphorylation of EGFR (**A**), AKT (**B**), MEK1/2 (**C**) and ERK1/2 (**D**). The experiments were repeated three times with similar results. Right panels represent densitometry analysis of western blots in (**A–D**). Densitometry data represent the mean ± SD from all three experiments. *p<0.05 when PCN-exposed cells were compared with the control cells exposed to same volume of sterile H_2_O.

Previous studies have shown that hyperoxic conditions activate the EGFR signaling pathway, leading to nuclear translocation of NRF2, and the transcription of ARE-containing antioxidant genes [33], [34]. Most recently, we and others have shown that PCN induces GCHM and the biosynthesis of major airway mucins MUC5AC and MUC5B in mouse airways, in part, by inducing the ROS-dependent EGFR signaling [11], [15]. We compared the levels of phosphorylated EGFR in A549 cells after 12 h of exposure to sterile H_2_O or various concentrations of PCN. Western blot analysis indicated that PCN at between 1.3 to 25 µg/ml increased the levels of phosphorylated EGFR between 1.4 - to 2-fold (Figure 6A). Furthermore, PCN exposure induced the phosphorylation of downstream components of the EGFR signaling cascade (Figure 6B, 6C, 6D). For example, after 12 hr of exposure, PCN (12.5 µg/ml) induced 4.0, 4.1 and 3.5-fold increases in p-AKT, p-MEK1/2 and p-ERK1/2, respectively (Figure 6B, 6C, 6D). Taken together, these results suggest that PCN activate the signaling of EGFR and its downstream PI3K-AKT/MEK1/2-ERK1/2 MAP kinase cascade, triggering the nuclear translocation of NRF2.

### EGFR, PI3K AKT and ERK1/2 Signaling Pathway Inhibitors Diminish PCN-induced NRF2 Accumulation in the Nucleus

**Figure 7:**
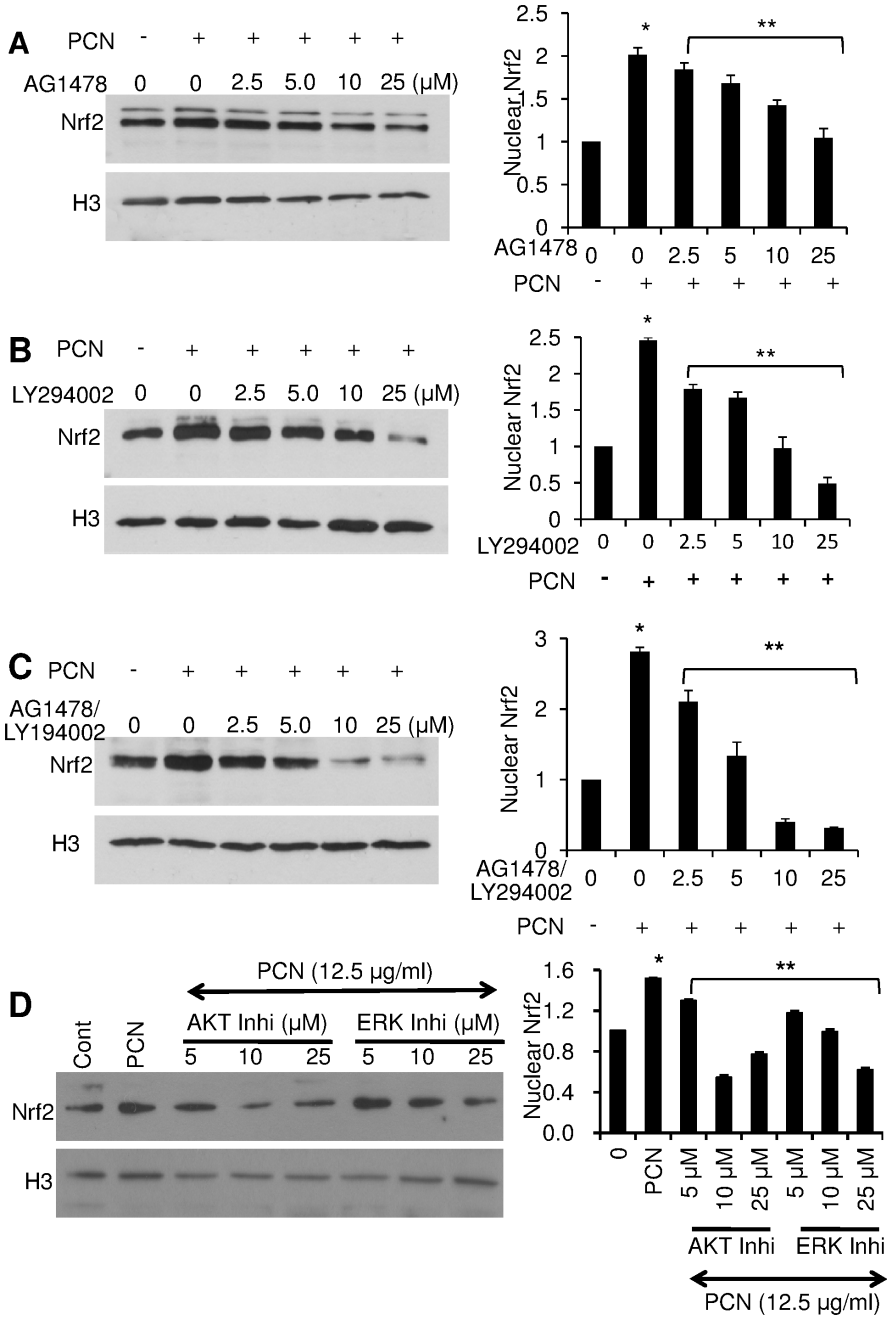
EGFR and PI3K inhibitors diminish PCN-enhanced NRF2 accumulation in the nuclei of A549 cells. A549 cells were exposed to the EGFR inhibitor AG1478 (**A**), the PI3K inhibitor LY294002 (**B**), or a combination of both AG1478 and LY294002 (**C**), or AKT or ERK inhibitors (**D**) for 90 min before the addition of PCN (12.5 µg/ml) for 12 hr. The expression of nuclear NRF2 was examined by monoclonal anti-NRF2 antibody and quantified by densitometry. The experiments were repeated three times with similar results. Densitometry data represent the mean ± SD from all three experiments. *p<0.05 when cells that were exposed to PCN were compared to control cells exposed to the same volume of sterile water. **p<0.05 when cells that were exposed to AG1478 or LY294002 or AG1478 plus LY294002, or AKT and ERK inhibitors were compared to cells exposed to PCN alone.

To further address whether the EGFR-PI3K-AKT/MEK1/2-ERK1/2 signaling pathway act as the upstream activator that induces NRF2-ARE-mediated transcriptional response to PCN, we examined the nuclear translocation of NRF2 in A549 cells in the presence or absence of the EGFR specific inhibitor AG1478, PI3K-specific inhibitor LY294002, ERK1/2 inhibitor 3-(2-Aminoethyl)-5-((4-ethoxyphenyl)methylene)-2,4-thiazolidinedione, and AKT Inhibitor VIII trifluoroacetate salt hydrate. The EGFR inhibitor AG1478 at concentrations of 2.5, 5.0, 10 and 25 µM significantly reduced the PCN-mediated translocation of NRF2 from the cytoplasm to the nucleus. In the absence of AG1478, the nuclear-localized NRF2 increased by 2-fold after 12 hr of exposure to PCN (12.5 µg/ml) (Figure 7A). In contrast, cells treated with 2.5, 5.0, 10 and 25 µM EGFR inhibitor AG1478 diminished PCN-enhanced NRF2 accumulation in the nuclei by 8%, 15%, 30% and 47.5%, respectively (Figure 7A). Similarly in the absence of PI3K inhibitor LY294002, nuclear-localized NRF2 was increased by 2.5-fold after 12 hr of exposure to PCN (12.5 µg/ml) (Figure 7B). In contrast, in the presence of 2.5, 5.0, 10 and 25 µM LY294002, the PCN-mediated NRF2 accumulation in the nuclei decreased by 27%, 32%, 60.6% and 80%, respectively. Next, we examined whether a combination of both AG1478 and LY294002 would allow a more robust inhibition of PCN-mediated NRF2 accumulation in the nuclei. The western blotting results showed that a combination of both inhibitors at the concentration of 2.5, 5.0, 10 and 25 µM, attenuated PCN-mediated NRF2 accumulation in the nuclei by 25%, 53%, 85.7% and 90%, respectively (Figure 7C). Finally, addition of both AKT and ERK1/2 inhibitors also significantly reduced the NRF2 nuclear translocation during PCN exposure (Figure 7D). Collectively, these results suggest that EGFR-PI3K-dependent signaling mediates the nuclear translocation of NRF2 in lung epithelial cells during PCN-mediated oxidative stress.

### EGFR Inhibitor and Antioxidant GSH Prevent the Inhibition of PCN-mediated Activation of AKT and ERK Signaling

**Figure 8:**
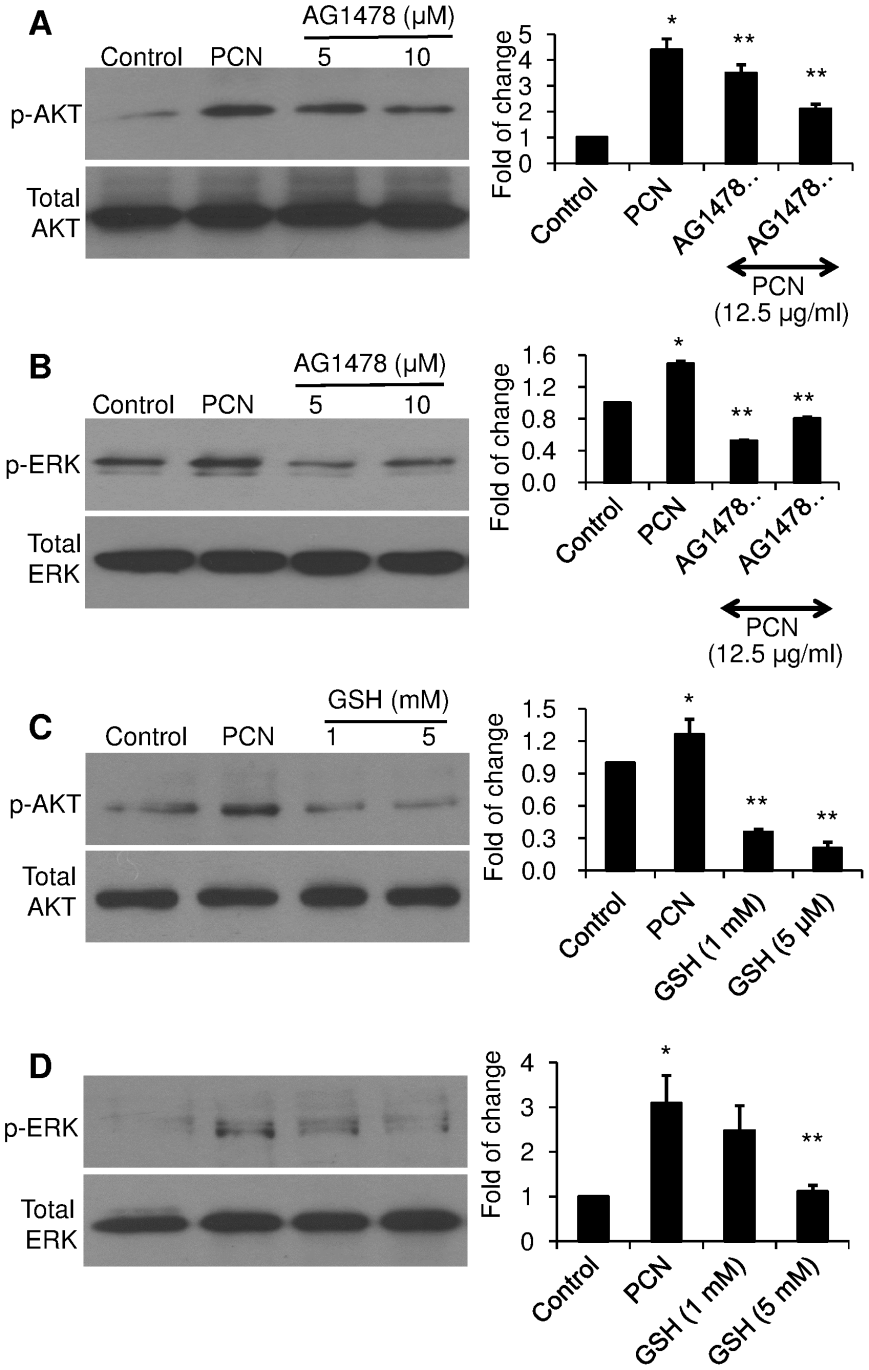
EGFR inhibitor and antioxidant GSH prevent the inhibition of PCN-mediated activation of AKT and ERK1/2 signaling. A549 cells were exposed to the EGFR inhibitor AG1478 (**A–B**) or GSH (**C–D**) for 90 min before the addition of PCN (12.5 µg/ml) for 12 hr. Control cells were exposed to same volume of sterile H_2_O. Cytoplasmic proteins were probed with anti-phospho–specific antibodies. (**A–B**) The phosphorylation of AKT and ERK1/2 in A549 cells treated with AG1478 and PCN. (**C–D**) The phosphorylation of AKT and ERK1/2 in A549 cells treated with GSH and PCN. Right panels represent densitometry analysis of western blots. The experiments were repeated three times with similar results. Densitometry data represent the mean ± SD from all three experiments. *p<0.05 when cells that were exposed to PCN were compared to control cells exposed to the same volume of sterile water. **p<0.05 when cells that were exposed to AG1478 or GSH were compared to cells exposed to PCN alone.

To provide a clearer demonstration AKT and ERK1/2 activation is downstream of PCN-induced EGFR activation, we examined whether inhibition of EGFR would block the activation of AKT and ERK. The induction of AKT (Figure 8A) and ERK1/2 (Figure 8B) phosphorylation by PCN were reduced in the presence of the EGFR inhibitor AG1478. These results confirm that PCN activates both AKT and ERK1/2 through the induction of EGFR signaling. Furthermore, provision of antioxidant GSH also blocked the phosphorylation of AKT and ERK1/2 by PCN (Figure 8C, 8D), suggesting that PCN-generated ROS were the indirect activators of EGFR. Collectively, our results indicate that PCN-generated ROS induce the EGFR-PI3K-AKT/MEK1/2-ERK1/2 signaling pathway, resulting in the nuclear translocation of NRF2 to activate the transcription of the antioxidative genes γ*GCSh* and *NQO1*.

## Discussion

PCN is a redox-active toxin secreted by *PA* that oxidatively damages pulmonary epithelial cells and is important for acute and chronic lung infections [4], [5], [10], [11], [35]. In contrast, NRF2 is crucial in cytoprotection by inducing the transcription of antioxidative and detoxifying enzymes through its binding to the promoter of genes containing the cis-acting ARE [36]. In this study, we demonstrate that PCN activates the EGFR-PI3K-AKT/MEK1/2-ERK1/2 signaling pathway, leading to increased nuclear translocation of NRF2, with corresponding increase in the transcription of ARE-containing *γ-GCSh* and *NQO1* genes in the lung epithelial cells. Three lines of evidence support these conclusions: (i) Western blotting and immunofluorescence analyses show that clinically-relevant concentrations of PCN induce the translocation and accumulation of NRF2 from cytoplasm to nuclei of lung epithelial cells at both concentration and time-dependent manners; (ii) Chronic exposure to PCN enhances nuclear translocation of NRF2 in airway epithelial cells developing GCHM and mucus hypersecretion; (iii) Increased levels of nuclear NRF2 are correlated with enhanced expression of two NRF2-ARE controlled antioxidant enzymes, NQO1 and γ-GCSh. Collectively, these data indicate that NRF2 plays important roles in mediating resistance to PCN-mediated oxidative stress.

We investigated the signaling pathway induced by PCN that triggers the nuclear NRF2 accumulation, leading to upregulation of antioxidative machinery that detoxify ROS. Several recent reports support a role for the EGFR-PI3K-AKT/MEK1/2-ERK1/2 pathway in regulating nuclear accumulation of NRF2 in response to oxidative stress. For example, in the murine alveolar epithelial cell line C10, inhibition of NADPH oxidase or the ERK1/2 pathway prevents hyperoxia-induced NRF2 translocation and ARE-dependent transcriptional activation [26], [34]. In addition, the activation of NRF2 after hyperoxic insult was not observed in ERK1-deficient cells [34]. These studies propelled us to examine whether PCN-mediated oxidative stress activates NRF2 nuclear translocation through the EGFR-PI3K-AKT/MEK1/2-ERK1/2 signaling pathway. Our results reveal that PCN increases the phosphorylated forms of EGFR, PI3K, AKT, MEK1/2 and ERK1/2, which correlate with the timing of NRF2 nuclear localization. Nuclear NRF2 accumulation increases the transcription of the genes encoding the antioxidant enzymes NQO1 and γ-GCSh. Consistent with these observations, both of EGFR and PI3K inhibitors block PCN-induced NRF2 accumulation in the nuclei of A549 cells. These experimental findings suggest that EGFR-PI3K-AKT/MEK1/2-ERK1/2 signaling pathway acts as one of the main signal transduction pathways triggering NRF2 activation and subsequent NRF2-ARE-mediated transcription of antioxidant genes in response to PCN-mediated oxidative stress.

Activation of the EGFR pathway by PCN-generated ROS appears to have driven the increased nuclear localization of NRF2 in the goblet cells within the mouse airways developing GCHM and mucus hypersecretion as a result of chronic exposure to PCN. This is consistent with previous finding by us and others, which shows that PCN causes GCHM and overexpression of two major airway mucins MUC5B [11] and MUC5AC [15] by activating EGFR. Mechanistically, PCN-activated EGFR signaling inhibits the expression of FOXA2, a major transcriptional repressor of GCHM and mucin biosynthesis [11]. Surprisingly, we did not observe statistically significant changes in the nuclear translocation of NRF2 in an acute model (1, 3, 6, 12 and 24 hr) after a single dose of PCN (25 µg in 50 µl) exposure (data not shown). It is possible that the immunofluorescence method was not sensitive enough to detect subtle transient differences in the amounts of NRF2 translocated into nuclei in this model of exposure.

Finally, we propose a possible model to illustrate the activation of NRF2 by PCN. PCN-generated ROS activate EGFR. Subsequently, EGFR activates PI3K, which phosphorylates AKT and MEK1/2-ERK1/2, respectively. Activated AKT and MEK1/2-ERK1/2 phosphorylate NRF2, causing the dissociation of NRF2:Keap1 complex in the cytoplasm, ultimately leading to the translocation of NRF2 to the nucleus, where it activates the transcription of ARE-containing genes that encode antioxidative and detoxification enzymes. Thus, therapeutic strategies prolonging or enhancing the expression of NRF2 may be beneficial in improving the lung function of patients with chronic PA infection, where PCN plays a prominent virulence role.

## Supporting Information

Figure S1 **PCN increases ROS production in airway epithelial cells.** **(A)** A549 cells were exposed to clinically-relevant concentrations of PCN for 12 hr and the total ROS levels were measured. The experiments were performed in triplicates and repeated three times with similar results. Representative results from one set of experiments are shown. *p<0.05 when PCN-exposed cells were compared with the control cells exposed to same volume of sterile water. **(B)** The viability of A549 cells exposed to indicated concentrations of PCN as determined by Trypan blue staining. Two hundred cells were counted for each sample.
(TIF)

Figure S2 **Nuclear translocation of NRF2 is increased in PCN-mediated goblet cell hyperplasia in mouse airways.** Mouse lungs (groups of 8) were exposed to sterile water control (**A**) or PCN (**B**). Lung sections were stained with an anti-NRF2 primary antibody and visualized with a goat anti-Rabbit DyLight® 488-conjugated secondary antibody. Nuclei were stained with DAPI. Images were photographed using confocal fluorescence microscope. Magnification: 40X. **A** and **B** show merged images of NRF2 and DAPI. Pink arrows indicate nuclei inside goblet cells obscured by NRF2. White arrows indicate alveolar epithelial cells largely clear of NRF2.
(TIF)
