# Supplementary figures and images for: Pseudomonas aeruginosa Pyocyanin Activates NRF2-ARE-Mediated Transcriptional Response via the ROS-EGFR-PI3K-AKT/MEK-ERK MAP Kinase Signaling in Pulmonary Epithelial Cells
Source: PLoS One. 2013 Aug 27;8(8):e72528. doi: 10.1371/journal.pone.0072528 (PMC3755003; doi:10.1371/journal.pone.0072528)

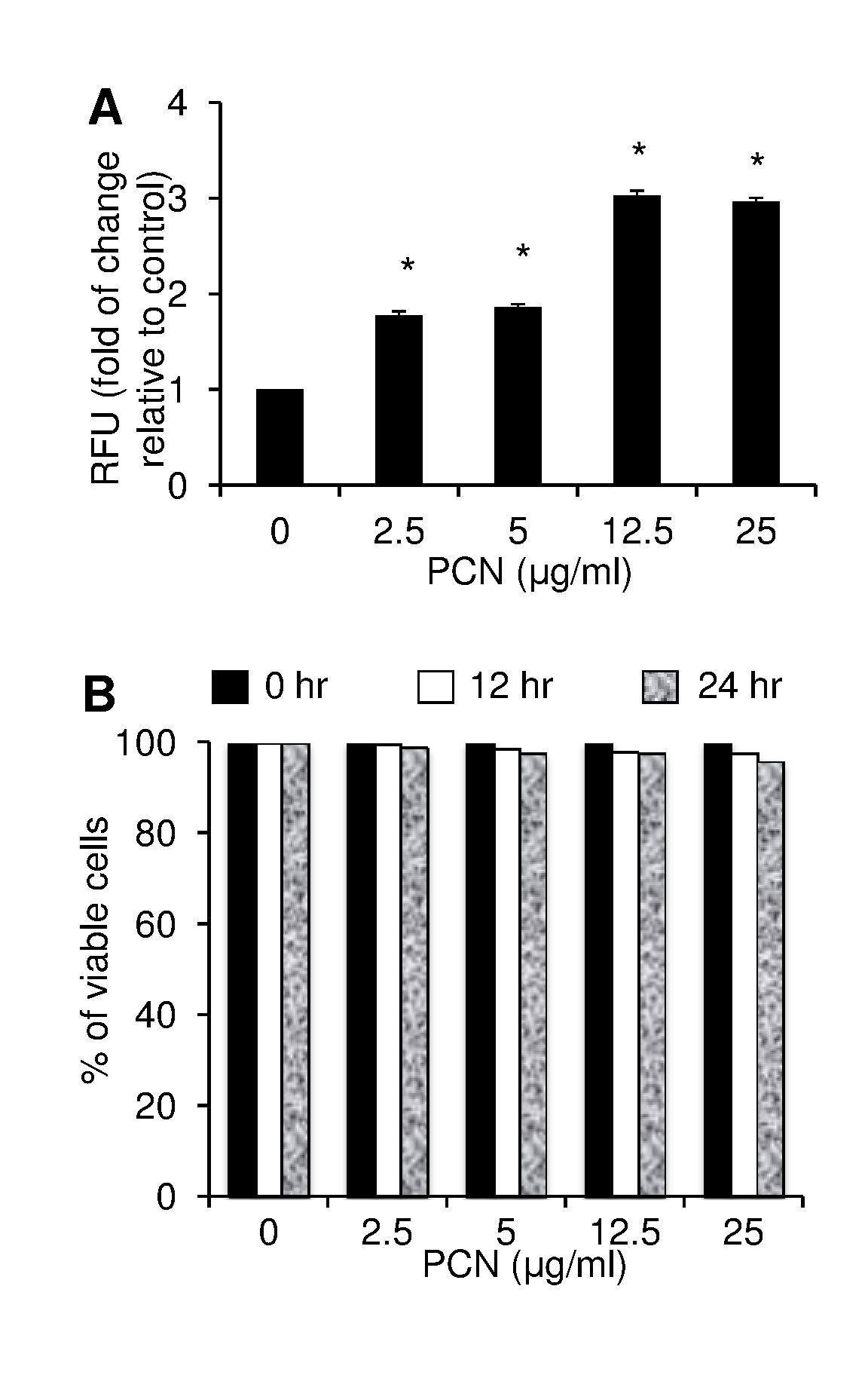

Supplement: Figure S1 — PCN increases ROS production in airway epithelial cells. (A) A549 cells were exposed to clinically-relevant concentrations of PCN for 12 hr and the total ROS levels were measured. The experiments were performed in triplicates and repeated three times with similar results. Representative results from one set of experiments are shown. *p<0.05 when PCN-exposed cells were compared with the control cells exposed to same volume of sterile water. (B) The viability of A549 cells exposed to indicated concentrations of PCN as determined by Trypan blue staining. Two hundred cells were counted for each sample. (TIF) [file pone.0072528.s001.tif]

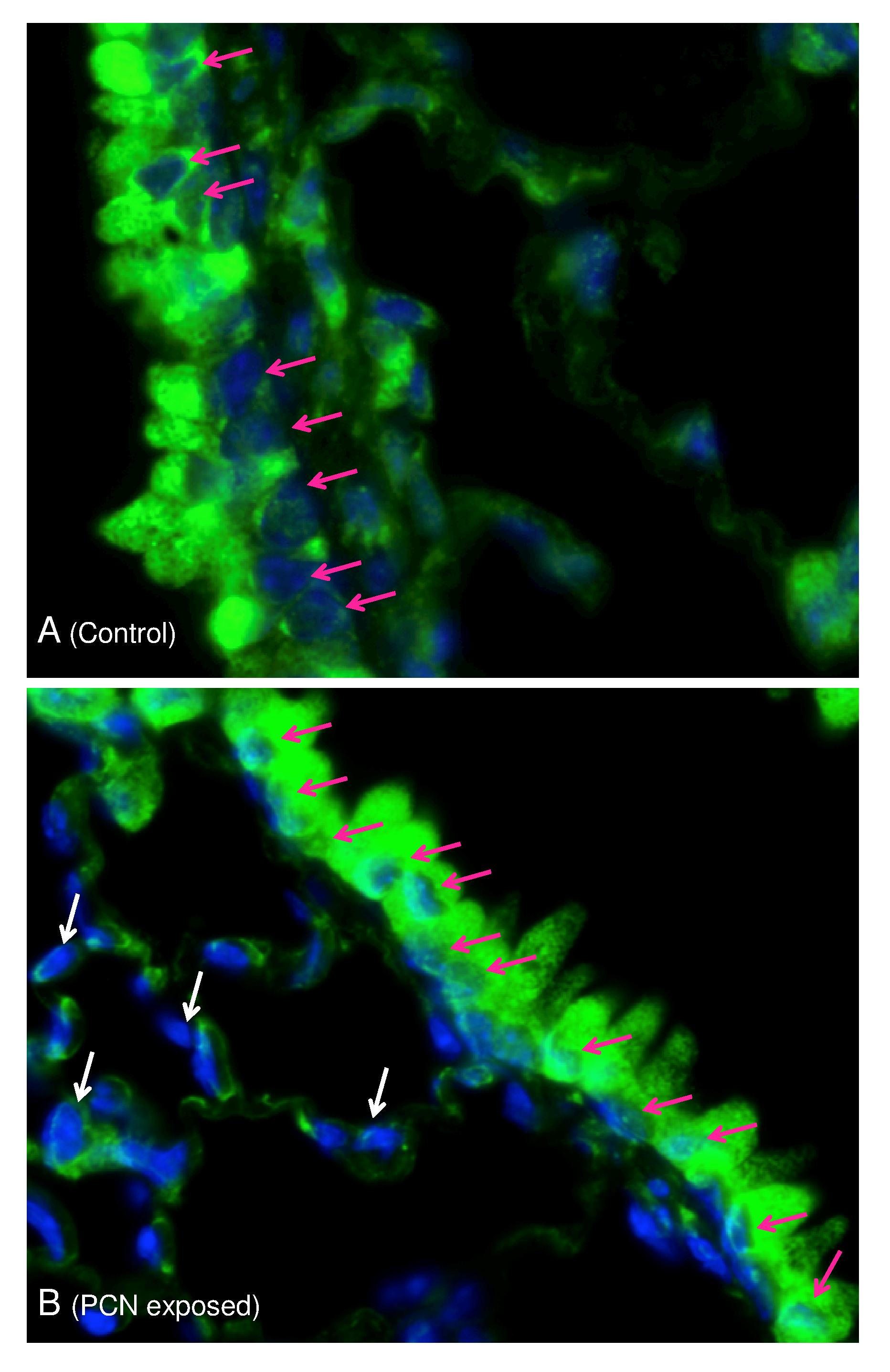

Supplement: Figure S2 — Nuclear translocation of NRF2 is increased in PCN-mediated goblet cell hyperplasia in mouse airways. Mouse lungs (groups of 8) were exposed to sterile water control (A) or PCN (B). Lung sections were stained with an anti-NRF2 primary antibody and visualized with a goat anti-Rabbit DyLight® 488-conjugated secondary antibody. Nuclei were stained with DAPI. Images were photographed using confocal fluorescence microscope. Magnification: 40X. A and B show merged images of NRF2 and DAPI. Pink arrows indicate nuclei inside goblet cells obscured by NRF2. White arrows indicate alveolar epithelial cells largely clear of NRF2. (TIF) [file pone.0072528.s002.tif]
